# Role of Oxidative Stress and Inflammation in Age Related Macular Degeneration: Insights into the Retinal Pigment Epithelium (RPE)

**DOI:** 10.3390/ijms26083463

**Published:** 2025-04-08

**Authors:** María Elena Ochoa Hernández, Lidianys María Lewis-Luján, María Guadalupe Burboa Zazueta, Teresa Del Castillo Castro, Enrique De La Re Vega, Juan Carlos Gálvez-Ruiz, Sergio Trujillo-López, Marco Antonio López Torres, Simon Bernard Iloki-Assanga

**Affiliations:** 1 Department of Scientific and Technological Research, University of Sonora, Luis Encinas y Rosales, Centro, Hermosillo 83000, Sonora, Mexico; aneleochoa@gmail.com (M.E.O.H.); maria.burboa@unison.mx (M.G.B.Z.); enrique.delare@unison.mx (E.D.L.R.V.); marco.lopez@unison.mx (M.A.L.T.); 2 Department of Biological Chemical Sciences, University of Sonora, Luis Encinas y Rosales, Centro, Hermosillo 83000, Sonora, Mexico; lidianys.lewis@unison.mx (L.M.L.-L.); juan.galvez@unison.mx (J.C.G.-R.); 3 Department of Research in Polymers and Materials, University of Sonora, Calle de la Sabiduría, Centro, Hermosillo 83000, Sonora, Mexico; teresa.delcastillo@unison.mx; 4 Department of Medicine and Health Sciences, University of Sonora, Luis Encinas y Rosales, Centro, Hermosillo 83000, Sonora, Mexico; sergio.trujillo@unison.mx

**Keywords:** age-related macular degeneration (AMD), oxidative stress, inflammation, retinal pigment epithelium (RPE), immunosenescence, bioinformatics

## Abstract

Age-related macular degeneration (AMD) is a leading cause of visual impairment worldwide, characterized by the accumulation of extracellular drusen deposits within the macula. The pathogenesis of AMD is multifactorial, involving oxidative stress, chronic inflammation, immune system dysregulation, and genetic predisposition. A key contributor to disease progression is the excessive accumulation of reactive oxygen species (ROS), which damage retinal pigment epithelium (RPE) cells and disrupt cellular homeostasis. Additionally, immunosenescence and chronic low-grade inflammation exacerbate AMD pathology, further impairing retinal integrity. Despite ongoing research, effective therapeutic options remain limited, and there is no definitive cure for AMD. This review explores the intricate molecular mechanisms underlying AMD, including the role of oxidative stress, chronic inflammation, and genetic factors in RPE dysfunction. Furthermore, we highlight potential therapeutic strategies targeting these pathways, as well as the emerging role of bioinformatics and artificial intelligence in AMD diagnosis and treatment development. By improving our understanding of AMD pathophysiology, we can advance the search for novel therapeutic interventions and preventative strategies.

## 1. Age-Related Macular Degeneration (AMD)

Age-related macular degeneration (AMD) has emerged as one of the leading causes of visual impairment worldwide. By 2040, its prevalence is projected to increase significantly, potentially impacting approximately 288 million individuals worldwide [1]. This condition is characterized by the presence of drusen in the macula, which are yellow extracellular deposits rich in lipoproteins. The number and size of the drusen found in the macula are closely associated with the progression of the disease [2,3]. Drusen forms when the lysosomal capacity of retinal cells diminishes. While lipids like cholesterol and phosphatidylcholine are their primary components, drusen also contains varying amounts of protein, zinc, carbohydrates, and components from the complement system [4]. The macula is a highly specialized region of the retina, densely populated with cone photoreceptors, which account for approximately 5% of all photoreceptors in the retina but are crucial for color vision and high-acuity central vision. These cones are concentrated in the fovea, the central portion of the macula, where they provide the sharpest visual resolution. Damage to cone photoreceptors, as observed in AMD, directly impacts these functions, leading to significant visual impairment. Furthermore, the dependency of cones on the surrounding retinal pigment epithelium (RPE) for nutrient exchange and phototransduction maintenance underscores their vulnerability to oxidative stress and chronic inflammation, key factors in AMD pathophysiology [5].

The human macula possesses two kinds of photoreceptors: cones and rods. Rods have a higher concentration than cones, and their function is vision in the dim light and color vision due to rhodopsin and opsins, which are pigments that hold distinct spectral absorptions. Now, little is known about cones since the study of them is more challenging, considering that mice only have 3% of them. The center of the macula has a rich concentration of cones, and these are dependent on the RPE for metabolic support, photopigment regeneration and phagocytosis and therefore, are at higher risk to be severely damaged by conditions such as AMD and high myopia, since they attack mainly the RPE (which is the cone environment) [5,6,7].

Cones are concentrated in the fovea, the central portion of the macula, where they provide the sharpest visual resolution. Damage to cone photoreceptors, as observed in AMD, directly impacts these functions, leading to significant visual impairment. Furthermore, the dependency of cones on the surrounding retinal pigment epithelium (RPE) for nutrient exchange and phototransduction maintenance underscores their vulnerability to oxidative stress and chronic inflammation, key factors in AMD pathophysiology [8].

**Figure 1:**
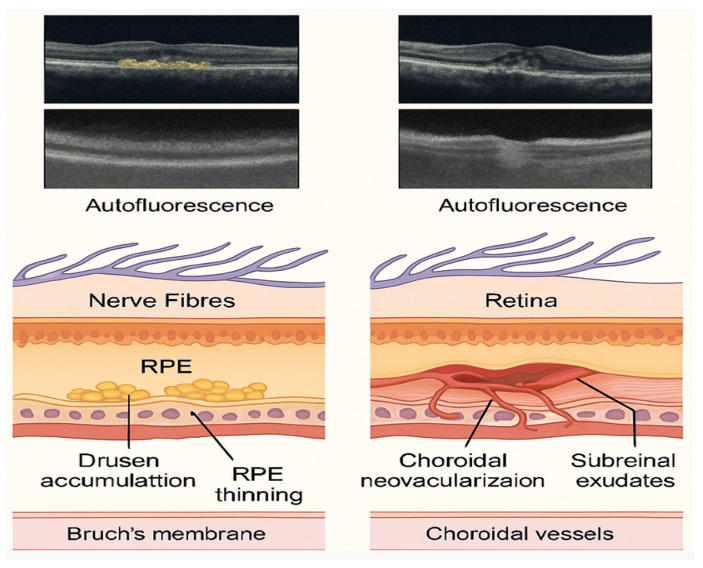
Comparative retinal changes in dry and wet age-related macular degeneration (AMD), including clinical imaging features. Cross-sectional schematic illustrations of the retina highlight characteristic findings in both dry and wet forms of AMD. On the left, dry AMD is represented by drusen accumulation beneath the retinal pigment epithelium (RPE) and progressive RPE thinning. On the right, wet AMD is shown with choroidal neovascularization penetrating through Bruch’s membrane and leading to subretinal exudates. Below each schematic, representative optical coherence tomography (OCT) and fundus autofluorescence images illustrate typical imaging features observed in clinical settings—such as RPE disruption, fluid accumulation, and reflectivity changes—helping to differentiate AMD stages for diagnosis and monitoring.

Clinically, age-related macular degeneration (AMD) manifests as a progressive deterioration of the macula, with significant implications for central vision and visual acuity. Advanced stages of AMD can be classified into two primary forms: exudative (wet) and dry. The wet form of AMD, also known as neovascular AMD, is primarily characterized by the growth of choroidal neovascularization, which can develop underneath or within the retina. It constitutes 10–20% of AMD cases but is responsible for most severe vision impairments. In contrast, the dry form of AMD involves the progressive atrophy of the retinal pigment epithelium (RPE) and the photoreceptors above it, leading to geographic atrophy. It accounts for approximately 80–90% of AMD cases and progresses slowly, leading to gradual vision loss [3]. Understanding these clinical presentations aids in distinguishing between disease stages and optimizing therapeutic interventions, particularly anti-VEGF therapies for wet AMD and potential future options for dry AMD. The damage resulting from both stages of AMD is illustrated in Figure 1.

Age is the primary factor in the development of AMD. However, lifestyle choices such as smoking, lack of physical activity, Western customs, ethnicity, and comorbidities have also been linked to the pathophysiology of AMD [9]. Increasing experimental evidence highlights the role of these modifiable risk factors in accelerating disease onset and progression. Smoking, one of the most well-established environmental risk factors, exacerbates oxidative stress in retinal tissues by reducing antioxidant defenses and enhancing the production of reactive oxygen species (ROS). In vivo studies have demonstrated that chronic exposure to cigarette smoke leads to degeneration of the retinal pigment epithelium (RPE), accumulation of drusen-like deposits, and progressive visual decline [10,11]. Similarly, physical inactivity contributes to vascular insufficiency and chronic inflammation, while regular physical activity has been shown to improve retinal perfusion and reduce systemic levels of pro-inflammatory cytokines, offering potential protective effects against AMD progression [9]. Dietary habits also play a crucial role; adherence to a Western diet—characterized by high intake of saturated fats and low antioxidant content—is associated with increased AMD risk, whereas antioxidant-rich diets, such as the Mediterranean diet, have been linked to improved retinal health and a reduced incidence of AMD. Collectively, these experimental findings underscore the importance of lifestyle modifications in AMD prevention and management. In addition to environmental and lifestyle factors, genetics play a significant role in this pathology, with studies showing that they can contribute up to 70% to the development of the disease. Currently, the complement factor H (CFH) gene is known to be directly associated with the disease through various pathways, including the negative regulation of pro-inflammatory activity [12,13].

Age-related macular degeneration (AMD) is a multifactorial disease influenced by genetic, environmental, and biological factors, with oxidative stress and chronic inflammation playing central roles in its pathogenesis [9,14]. The accumulation of reactive oxygen species (ROS) and immune dysregulation in the retinal pigment epithelium (RPE) stem from oxidative stress, which is defined as an imbalance between ROS production and elimination [15]. This imbalance contributes to cellular damage and neurodegeneration, ultimately leading to vision loss. However, despite significant advancements in understanding these mechanisms, a major challenge remains in identifying precise molecular pathways that drive AMD progression and developing targeted interventions. This gap has led to an increased reliance on bioinformatics and computational modeling to decipher complex interactions between oxidative stress, inflammation, and genetic predisposition in AMD.

This review aims to provide a cutting-edge, integrative perspective on the interplay between oxidative stress, chronic inflammation, and bioinformatics in the pathogenesis of age-related macular degeneration (AMD). By bridging molecular biology, genomics, and computational approaches, we seek to uncover novel insights into disease mechanisms, identify potential therapeutic targets, and highlight the role of artificial intelligence in predicting disease progression. This synthesis will offer a transformative framework for future research, paving the way for precision medicine strategies that could revolutionize AMD diagnosis and treatment.

## 2. Oxidative Stress

Reactive oxygen species (ROS) are highly reactive molecules characterized by unpaired electrons in their outer orbitals. Common examples of ROS include peroxides, hydroxyl radicals, and superoxide. These molecules can originate from both internal sources, such as by-products of mitochondrial metabolism, and external sources. In a balanced system, ROS serves a specific role as signaling molecules, which can be neutralized by antioxidants and antioxidant enzymes [16]. Examples of endogenous reactive oxygen species (ROS) sources include NADPH oxidases found in endothelial cell membranes and cytochrome P450 oxygenase, lipoxygenase, and cyclooxygenase. Externally, smoking, UV radiation, fatty acids, and certain metals contribute to ROS production, as well as to the formation of lipid peroxides, which may damage the cell membrane [10].

**Figure 2:**
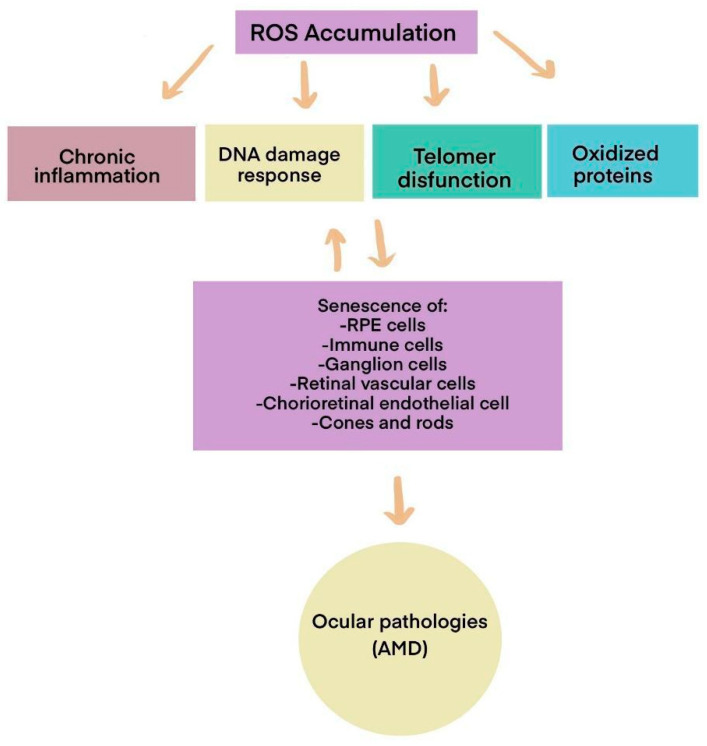
Visual scheme of ROS-induced damage in retinal cells (modified from Chan, T.C. et al., 2022 [10]).

While we naturally possess defenses against reactive oxygen species (ROS), these antioxidant defenses decline with age. As a result, ROS accumulates, leading to cellular dysfunction in various organs by oxidizing macromolecules such as lipids, proteins, and nucleic acids, as illustrated in Figure 2.

Mitochondria produce approximately 90% of cellular reactive oxygen species (ROS), with older cells often generating even more than younger ones. This production occurs through the electron transport chain. Additionally, ROS are produced during the phagocytosis of the outer segments of photoreceptors, primarily due to hydrogen peroxide (H_2_O_2_) generated by NADPH oxidase. Furthermore, β-oxidation of outer-segment lipids in peroxisomes also contributes to ROS production. Other endogenous sources of ROS include various enzymes, such as cyclooxygenases, xanthine oxidases, cytochrome P450 enzymes, and lipoxygenases [17].

Oxidative stress and chronic inflammation in AMD create a feedback loop that exacerbates retinal damage. ROS accumulation impairs mitochondrial function in RPE cells, leading to the release of inflammatory mediators, such as interleukin-6 (IL-6), tumor necrosis factor-alpha (TNF-α), and complement system activation. This pro-inflammatory microenvironment, in turn, amplifies oxidative stress by recruiting immune cells that further produce ROS, ultimately accelerating photoreceptor degeneration [10,15,17]. Recent transcriptomic analyses suggest that oxidative damage alters the gene expression profiles of RPE cells, triggering pathways associated with cellular senescence, complement dysregulation, and angiogenesis. However, understanding the precise genetic and molecular mechanisms behind these processes requires an integrative bioinformatics approach.

### 2.1. Cellular Senescence

**Figure 3:**
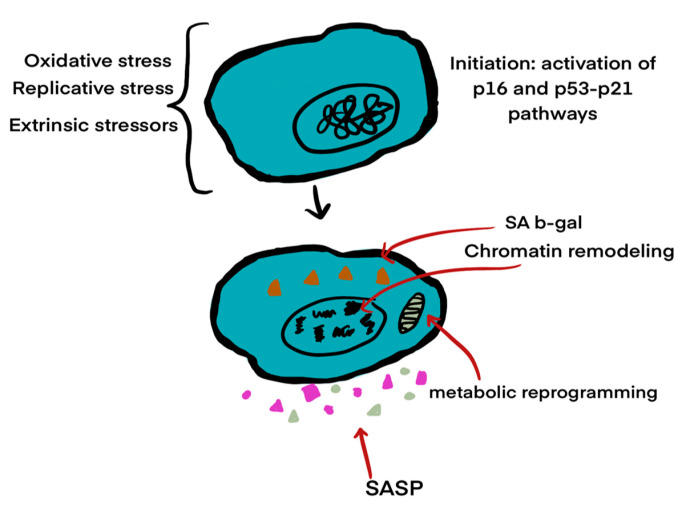
The three stages of cellular senescence, highlighting chromatin remodeling, DNA damage responses, and inflammatory secretory phenotypes (Modified from Blasiak, 2020 [24]).

The buildup of reactive oxygen species (ROS) causes cellular damage and can also result in cellular senescence (see Figure 3). Cellular senescence is characterized by a loss of proliferative capacity and resistance to apoptosis-inducing signals. Since senescent cells do not undergo apoptosis, they can accumulate over time [18]. This process leads to the senescence-associated secretory phenotype (SASP), marked by the secretion of pro-inflammatory cytokines, which leads to the release of inflammatory substances. These substances include pro-inflammatory cytokines, such as interleukin (IL)-1α, IL-1β, IL-6, and IL-8, along with chemokines like CXCL1 and CXCL2. Additionally, matrix metalloproteinases (MMPs) and inflammatory factors, such as vascular endothelial growth factor (VEGF), are secreted. These substances serve as modulators of the immune response and contribute to the changes observed in AMD [19,20]. In summary, releasing these pro-inflammatory signals triggers a physiological response aimed at eliminating pathogens and damaged cells. This reaction involves attacking the pathogens’ membranes and further promoting inflammation [21].

Understanding the interaction between VEGF and immune responses is essential, as VEGF is the primary inducer of angiogenesis. It modulates protein expression in endothelial cells and affects vascular permeability, which can occur through direct or indirect mechanisms [22]. VEGF is also found in other immune cells, such as T and myeloid cells, which can influence their phenotype and function [23].

Cellular senescence is a state of irreversible growth arrest triggered by various stressors, including oxidative damage, mitochondrial dysfunction, and DNA damage. Senescent cells remain metabolically active but acquire a distinct secretory profile known as the senescence-associated secretory phenotype (SASP), characterized by the release of pro-inflammatory cytokines, chemokines, growth factors, and proteases [24]. In the context of AMD, accumulation of senescent retinal pigment epithelial (RPE) cells contributes to chronic inflammation and tissue remodeling. SASP factors can amplify oxidative stress, promote immune cell recruitment, and disrupt the extracellular matrix, thereby exacerbating the degenerative microenvironment of the macula [25]. The mechanism of senescence is illustrated in Figure 3.

Research has shown that different stress signals can trigger the formation of senescent cells. Various pathways may be involved in this cellular process, influenced by factors such as the duration from the initial stress, the specific location of the cells, and the type of cell responsible for their senescence. However, the p53/ARF and RB/16 tumor suppressor pathways are the most prominent. These pathways halt the cell cycle and induce senescence [26]. Senescence is involved in age-related macular degeneration (AMD) through several pathways: (1) response to oxidative stress; (2) damage to macular tissue; (3) degeneration of the choriocapillaris mediated by a membrane attack complex; (4) the cyclic GMP-AMP synthase stimulatory pathway of interferon genes (cGAS-STING); (5) the DNA damage response; and (6) the accumulation of amyloid beta peptides [24].

### 2.2. Identification of Gaps in the Field: Immunosenescence and Genetic Discrepancies in AMD

Another significant contributor to ROS accumulation is immunosenescence, a process that typically begins around age 50. This age-related decline in both innate and adaptive immune functions reduces autophagy and increases susceptibility to infections, autoimmunity, cancer, and chronic inflammation [27]. Senescent immune cells accumulate in various tissues, including the brain, where they trigger inflammatory responses and disrupt iron metabolism—mechanisms associated with neurodegeneration and cognitive decline [28]. Chronic inflammation and immunosenescence are closely linked to the development of multiple age-related diseases, including AMD [29]. Although complex, the role of systemic inflammation in AMD has been increasingly explored, with substantial evidence supporting its contribution to disease onset and progression. However, the specific impact of immune aging on retinal pigment epithelium (RPE) dysfunction, complement system dysregulation, and microglial activation remains an area that warrants further investigation.

**Figure 4:**
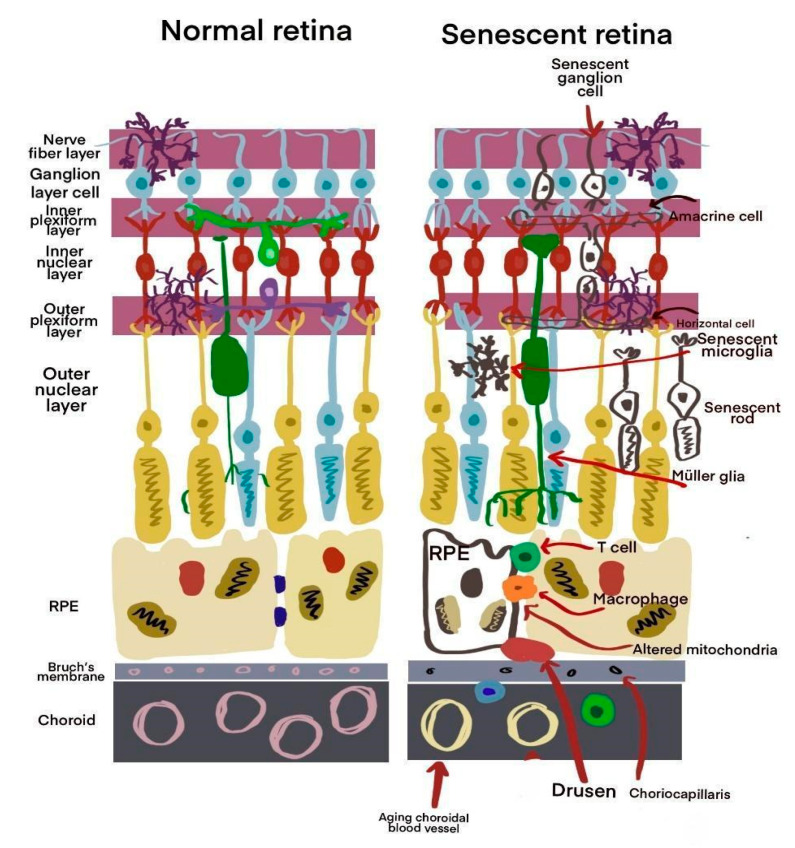
The contrast between a healthy retina and a senescent (aging) retina is significant. In a senescent retina, there are damaged cells and the presence of systemic immune cells that are not typically found there. Additionally, drusen, which are small, yellowish deposits, begin to accumulate. Bruch’s membrane becomes thicker, and the choriocapillaris and the vascular network are reduced. As a result, the number of vessels decreases in the aged choroid (Modified from Lee et al., 2021 [28]).

There is a known association between aging and chronic inflammation, leading to the concept of immunoaging. This term describes a persistent inflammatory state characterized by increased levels of cytokines and chemokines in tissues. This process is a natural part of aging and is crucial in immunosenescence, particularly in age-related pathologies such as age-related macular degeneration (AMD). Inflammation affects both adaptive and innate immune cells. For example, in macrophages, there is a reduction in phagocytic activity, which increases pro-inflammatory mediators in the retina and choroid [28]. Senescence damage is illustrated in Figure 4.

Studies suggest that aging-associated immune dysfunction alters cytokine production and reduces the efficiency of cellular repair mechanisms in the retina. However, current therapeutic approaches targeting inflammation in AMD do not specifically address immunosenescence-driven mechanisms. There is a growing need to develop interventions that modulate immune aging, such as senolytic drugs or immune-rejuvenating therapies, which could potentially alter the trajectory of AMD progression. Future research should focus on understanding how interventions targeting immunosenescence could complement existing AMD treatments, particularly in individuals with a higher genetic predisposition to chronic inflammation.

Inflammatory genes, such as interleukins, chemokines, complement factors, ARMS2, and NLRP3, play essential roles in the development of AMD [30]. The functions of interleukins 1, 6, and 8 have been previously discussed. Another important gene that has been extensively studied is complement factor H (CFH), which plays a vital role in the cleavage of C3 and the subsequent degradation of its products. Specific CFH subtypes, such as rs1061170 and rs141099, have been associated with a significantly increased risk of developing age-related macular degeneration (AMD). These subtypes also negatively affect the binding of heparin sulfate proteoglycans, leading to increased production of drusen in Bruch’s membrane. Additionally, mutations in the CFH gene are known to elevate the risk of developing AMD [12,13].

The complement system consists of numerous proteins found in plasma and on cell surfaces. These proteins are crucial in enhancing the body’s defense against pathogens. They identify and tag pathogens for destruction, initiate inflammation, help clear immune complexes, and form the membrane attack complex (MAC) to eliminate pathogens effectively. The complement system is also a vital link between innate and adaptive immunity [31]. Inflammation plays a crucial role in the development of AMD, with the complement system being significantly involved in the disease’s onset. When immune cells are recruited and activated, they release anaphylatoxins C3 and C5, which affect retinal microglia cells, lymphocytes, macrophages, monocytes, and mast cells. The most vital genetic link between AMD and the complement system has been identified in the CFH 1061170 gene variant [12,32]. Furthermore, persistent inflammation and dysregulation of the complement cascade within the retina are critical to drusen formation [31]. AMD genetic variants in complement genes C3, CFB, and CFH indicate that complement activation is driven by genetic status [9].

Genetic variants at the 10q26 locus—including ARMS2 and HTRA1—are significantly associated with AMD risk [33]. In contrast, the NLRP3 gene provides instructions for assembling a cytosolic multiprotein complex called an inflammasome. This inflammasome activates the protease caspase-1, which induces a type of programmed cell death called pyroptosis (dependent on gasdermin D) and enhances the release of interleukins 1β and 18. These processes contribute to innate immune defense and the maintenance of homeostasis. However, abnormal inflammasome activation is linked to various inflammatory diseases and can lead to cell death, including apoptosis, necroptosis, and ferroptosis [34].

Genetic predisposition is a significant contributor to AMD, accounting for up to 70% of disease susceptibility. Two major loci have been consistently identified as key genetic risk factors: chromosome 1q32, which includes the complement factor H (CFH) gene, and chromosome 10q26, which harbors the age-related maculopathy susceptibility 2 (ARMS2) and high-temperature requirement A serine peptidase 1 (HTRA1) genes. The CFH gene is central to the regulation of the complement system, particularly in inhibiting overactivation. Variants like rs1061170 (Y402H) increase AMD risk by impairing the protein’s ability to bind heparan sulfate and C-reactive protein, thereby promoting chronic inflammation and drusen formation in the retina. In contrast, ARMS2 and HTRA1 on chromosome 10q26 are associated with increased oxidative stress and dysregulated angiogenesis. The ARMS2 A69S polymorphism and HTRA1 promoter variants lead to altered protein expression and enhanced susceptibility to both the dry and neovascular forms of AMD [31,32,33].

Notably, these genetic loci influence disease progression differently. CFH variants are more closely linked to the early stages of AMD and drusen accumulation, while ARMS2 and HTRA1 variants are more strongly associated with the transition to advanced AMD, particularly the neovascular form [31,33]. Understanding the distinct contributions of these loci is essential for targeted therapies aimed at mitigating complement dysregulation and oxidative damage in AMD [12,13].

Although genetic studies have significantly advanced our understanding of AMD susceptibility, notable disparities exist in the expression and impact of genetic variants across different ethnic and geographic populations. Most genome-wide association studies (GWAS) and AMD-related genetic research have predominantly focused on populations of European descent, with limited representation of other ethnic groups. As a result, key genetic markers, such as CFH (rs1061170) and ARMS2/HTRA1 (10q26), may have varying levels of influence on AMD risk in non-European populations [35].

For instance, while CFH Y402H has been identified as a major risk allele in Caucasian populations, studies in East Asian cohorts suggest a lower frequency of this variant and a stronger association with other complement-related genes, such as C2 and CFB. Similarly, the role of ARMS2/HTRA1 in AMD susceptibility differs among populations, with some studies indicating variations in allele frequencies and disease association strength. These discrepancies highlight the necessity for more diverse genetic studies to refine risk prediction models and develop personalized medicine approaches for AMD [36].

Moreover, epigenetic modifications and gene–environmental interactions remain poorly understood in the context of AMD across different ethnic groups. Factors such as dietary patterns, exposure to environmental oxidative stressors, and socioeconomic determinants may influence genetic expression and AMD susceptibility. Addressing these gaps requires more inclusive research strategies, integrating multi-ethnic genetic databases and bioinformatics-driven analyses to unravel population-specific genetic risk factors [37].

To bridge these gaps, future research should conduct the following:

1. Investigate the specific molecular pathways through which immunosenescence contributes to AMD progression and assess the potential of senolytic and immune-modulating therapies.
2. Expand AMD genetic studies to underrepresented populations to better understand ethnic variations in disease susceptibility.
3. Integrate bioinformatics approaches, such as single-cell transcriptomics and machine learning models, to identify population-specific genetic and epigenetic markers.
4. Develop precision medicine strategies that consider genetic ancestry, immune aging, and environmental factors in tailoring AMD prevention and treatment.

By addressing these gaps, the field can move toward a more comprehensive and personalized understanding of AMD, ultimately improving disease management and therapeutic outcomes for diverse populations.

## 3. Retinal Pigment Epithelium (RPE)

Retinal pigment epithelium (RPE) cells play a crucial role in maintaining the health of photoreceptors and choriocapillaris. These cells are in direct contact with the outer segments of photoreceptors on their apical side and Bruch’s membrane on their basal side. RPE cells perform phagocytosis, helping to degrade the outer segments of photoreceptors. They also facilitate the entry of nutrients and the elimination of metabolic waste. Additionally, these cells are involved in vitamin A metabolism and light absorption, and they help maintain the external blood–retinal barrier. Each RPE cell supports approximately 40 photoreceptors, emphasizing their critical role in retinal function [19,38,39]. These cells are crucial in developing age-related macular degeneration (AMD).

Inflammation releases Toll-like receptors (TLRs), inflammasome receptors, and complement system components, which initiate a series of cell-signaling cascades [39,40]. Then, choroidal neovascularization is closely associated with inflammatory cytokines, complement system activation, and ongoing suppression of macrophages [41,42]. An inflammatory response triggers two types of immunity: adaptive and innate. In the retinal pigment epithelium (RPE) context, inflammation primarily involves innate immunity, specifically the complement system and the inflammasome. For instance, during the early stages of age-related macular degeneration (AMD), the RPE activates the inflammasome as an initial protective measure and recruits macrophages. These macrophages accumulate within Bruch’s membrane, enhancing inflammasome activity [41].

A study by Klettner et al. (2020) demonstrated that long-term inflammatory stimuli, such as Toll-like receptors (TLRs), lipopolysaccharide (LPS), and the pro-inflammatory cytokine TNFα, reduce the viability of retinal pigment epithelium (RPE) cells and alter their barrier properties [42]. These changes suggest a potential mechanism for age-related macular degeneration (AMD). The researchers also found that the loss of RPE65 results from prolonged pro-inflammatory signaling in the RPE, highlighting a possible pathway through which inflammation leads to RPE dysfunction and subsequent photoreceptor degeneration. TLRs are immune receptors that detect molecular patterns associated with microbial pathogens. When these pathogens are recognized, a signal transduction cascade is activated, resulting in a rapid inflammatory response characterized by cellular activation and the production of various cytokines, type 1 interferons, and chemokines. The literature indicates TLR3 activation in RPE cells is linked to RPE cell damage and may be associated with retinal degeneration in AMD and other retinal disorders [43,44].

Lee et al. (2021) [28] explain that pathological changes in the retinal pigment epithelium (RPE) in individuals predisposed to age-related macular degeneration (AMD) occur before the death of rod and cone photoreceptors. The functional deterioration of these cells leads to the retinal barrier breakdown between the retina and the choroid, which makes way for the development of drusen and macular damage. Additionally, human studies on senescent RPE cells have revealed a genetic signature of senescence in AMD, characterized by the upregulation of p16INK4a, p53, and bone morphogenetic protein (BMP4) [42].

The study by Macchioni et al. (2021) indicates that RPE cells are the primary contributors to the inflammatory environment in the aging human retina. While moderate inflammatory responses can help maintain homeostasis during healthy aging, excess inflammatory stimuli can increase the release of pro-inflammatory cytokines. This overactivity may be a link between chronic oxidation and harmful chronic inflammation, both of which are significant factors in the pathogenesis of AMD [45].

## 4. Angiogenesis

Angiogenesis and inflammation are distinct processes, yet they are interconnected because angiogenesis is directly related to inflammation in adult organisms [45]. Angiogenesis is a crucial and natural process necessary for wound healing. This intricate process ensures that healing tissues receive essential oxygen and nutrients. This repair mechanism encompasses several molecular methods [46]. Endothelial cells play a fundamental role in angiogenesis, making up the tunica intima of blood vessels. These cells bind to the soft muscle cells and pericytes found in the tunica media. Moreover, the tunica adventitia, which consists of connective tissue, collagen, and fibers, is formed around the blood vessels.

In the context of age-related macular degeneration (AMD), particularly the wet or neovascular form, abnormal changes occur in the morphology and function of blood vessels in the retina. The choroid, located at the back of the eye between the sclera and the retinal pigment epithelium (RPE), is a highly vascularized tissue responsible for supplying oxygen and nutrients to the RPE and neuronal cells, as well as facilitating the removal of cellular waste products. Any abnormalities in the choroid can lead to retinopathies, especially AMD [47]. The choroid supplies blood to the retinal pigment epithelium (RPE) and the retina. Abnormal growth of choroidal blood vessels can lead to a condition known as choroidal neovascularization (CNV). In severe cases of age-related macular degeneration (AMD), this can result in irreversible blindness. RPE cells produce angiogenic proteins, including factors that promote CNV, such as angiopoietin 2 (ANG2), vascular endothelial growth factor (VEGF), and fibroblast growth factor 2 (FGF2) [48].

The retinal pigment epithelium (RPE) secretes several growth factors that help regulate vascular endothelium homeostasis. One crucial factor is the pigment-epithelium-derived factor (PEDF), which acts as an antiangiogenic molecule in the choriocapillaris and provides neuroprotection against hypoxia-induced apoptosis. Additionally, RPE cells produce erythropoietin (EPO), which protects retinal cells from oxidative stress and light-induced damage by inhibiting enzyme caspase and preventing apoptosis. EPO also regulates the expression of proangiogenic factors such as vascular endothelial growth factor (VEGF) [49].

A study by Farjood et al. (2020) [49] demonstrated that acute mechanical stress triggers the expression of key angiogenic factors that promote angiogenesis in vitro. This suggests that mechanical stress contributes to angiogenesis in age-related macular degeneration (AMD). In this study, mechanical stress was applied to primary porcine retinal pigment epithelial (RPE) cells, and the expression of main angiogenic factors—such as VEGF, ANG2, IL-6, IL-8, and TNF-α—was analyzed using immunocytochemistry, quantitative reverse transcription polymerase chain reaction (qRT-PCR), and enzyme-linked immunosorbent assay (ELISA). Additionally, hypoxia-inducible transcription factors are significant in the induction of angiogenesis, as they upregulate VEGF production in response to hypoxic conditions [50,51].

There are several cells involved in retinal vascularization, such as astrocytes and Müller glial cells, which secrete factors like VEGF. It has been seen in diabetic retinopathy that pericytes are lost, contributing to the multiple vascular changes, including the loss of formed vessels, the loss of normally growing vessels, and the development of abnormal angiogenesis. Müller cells play a major role in the structure and function of the macula. Nonetheless, these cells also have a key role in the development of CNV lesions and RPE atrophy, secreting inflammatory cytokines and factors like PEDF, TSP-1, VEFG, Il-1B, TNF-a, and IL-6 [52,53]. The current treatment for AMD therapy includes intravitreal injections of VEGF-neutralizing agents, preventing the interaction between VEGF and its receptor (VEGFR-2), since the binding triggers the endothelial cell migration and proliferation. Even though it may have significant benefits it can bring, these drugs show unsatisfactory effects in a significant percentage of patients, recalling the need for additional treatment of neovascular AMD [54].

## 5. Bioinformatics Aspect of Gene Expression Trends in AMD

Bioinformatics approaches have significantly advanced our understanding of gene expression trends during AMD progression. Transcriptomic analyses of retinal tissue and retinal pigment epithelium (RPE) from AMD patients have revealed dynamic changes in the expression of genes involved in the complement cascade, oxidative stress response, and extracellular matrix remodeling. For instance, studies leveraging RNA sequencing and microarray data have consistently identified upregulation of complement genes, such as C3 and CFH, during the early stages of AMD, correlating with increased inflammation and drusen formation [55].

As AMD progresses, bioinformatics analyses have revealed a dynamic a shift in gene expression profiles, particularly toward pathways involved in angiogenesis (e.g., upregulation of VEGFA and HTRA1), lipid metabolism (APOE, ABCA1), and cellular senescence (CDKN2A). Among these, the dysregulation of lipid metabolism has emerged as a crucial contributor to AMD pathogenesis. Recent studies have emphasized the significant roles of lipid-related genes such as APOE, ABCA1, LIPC, and CETP, which are involved in lipid transport, cholesterol efflux, and lipoprotein remodeling within the retinal pigment epithelium (RPE) and Bruch’s membrane. These processes are closely linked to drusen formation and chronic inflammation [56,57].

Lipid metabolism genes such as APOE, ABCA1, LIPC, and CETP play fundamental roles in the pathogenesis of age-related macular degeneration (AMD) by influencing lipid transport, cholesterol efflux, and lipoprotein remodeling within the retinal pigment epithelium (RPE) and Bruch’s membrane. APOE isoforms, in particular, differentially modulate drusen formation and retinal degeneration. In vivo studies have shown that the APOE2 isoform increases susceptibility to AMD-like pathology, while APOE4 exhibits a protective effect against retinal damage and drusen accumulation [55]. ABCA1, a crucial cholesterol transporter in the RPE, maintains lipid homeostasis by facilitating cholesterol efflux. Its deficiency leads to lipid accumulation in Bruch’s membrane and functional impairment of the RPE, which are key features in AMD progression [56]. Meanwhile, LIPC and CETP, regulators of high-density lipoprotein (HDL)-mediated lipid transport and clearance, have been associated with altered lipid metabolism in the retina. Genetic association studies have linked dysregulation in these genes to an increased risk of AMD and enhanced lipid deposition in the subretinal space [55]. Together, these findings underscore the importance of lipid metabolic balance in maintaining retinal integrity and preventing degenerative changes characteristic of AMD.

In addition, LIPC and CETP, which regulate HDL metabolism, have been associated with altered retinal lipid profiles and increased susceptibility to AMD in genome-wide association studies. Collectively, these findings from both in vitro and in vivo studies indicate that impaired lipid clearance and disrupted cholesterol transport contribute to oxidative stress, chronic inflammation, and complement activation—hallmarks of AMD progression. Complementing these genetic insights, pathway enrichment studies using tools like Gene Ontology (GO) and Kyoto Encyclopedia of Genes and Genomes (KEGG) have highlighted oxidative phosphorylation and mitochondrial dysfunction as central themes in late-stage AMD. Moreover, single-cell RNA sequencing (scRNA-seq) has recently provided unprecedented resolution of cell-type-specific expression changes, uncovering interactions between stressed RPE cells, activated microglia, and infiltrating immune cells.

Integrating these bioinformatics insights not only elucidates the molecular mechanisms driving AMD but also offers potential biomarkers for early detection and progression monitoring. Furthermore, computational modeling of gene regulatory networks has been instrumental in predicting how specific genetic variants, such as those in CFH and ARMS2, alter downstream gene expressions, contributing to AMD pathophysiology [54]. The genetics of AMD have been studied worldwide, with the most common variants being associated with the cluster of genes near complement factor H (CFH) and complement factor H-related (CFHR) 5 on chromosome 1q32 (Chr1 locus). Along with these, age-related maculopathy susceptibility 2 (ARMS2) and high temperature requirement factor A1 (HTRA1), two strongly linked genes, are located on chromosome 10q26 (Chr10 locus) [56].

Even though these loci by themselves have a low impact on the incidence of AMD, the genetic variants within the 10q26 region of Chr10 and the 1q32 region of Chr1 together account for an up to 50% risk of developing AMD. Moreover, there are several coding and noncoding SNPs within region 10q26 as well as an insertion–deletion mutation in the ARMS2 3’ untranslated region; therefore, it has not been possible to determine which gene or variant within *Chr10* gives a higher risk to develop AMD. ARMS2/HTRA1 is also linked to a higher risk for neovascular AMD, more than CFH-CFHR5, where genetic experiments have suggested the genetic changes at ARMS2/HTRA1 affect the vascular wall and the RPE-Bruch membrane interface [56]. However, the 10q26 has been called an “enigma” by Merle et al. (2023) because the products of the targeting of this gene have a lack of functional data. They stated these gene variants as the strongest contributors to AMD genetic risk and have linked epigenetic alterations to the expression levels of 10q26 in diseased individuals. Nonetheless, this “enigma” may be solved by new AI tools [58].

Machine learning algorithms and artificial intelligence (AI) models have further enhanced our ability to predict disease trajectories by integrating patient-specific genetic data, environmental risk factors, and imaging biomarkers. AI-driven retinal imaging analyses now enable early AMD detection by identifying subtle retinal changes that precede clinical manifestations. Moreover, computational models of gene regulatory networks have been instrumental in predicting how oxidative-stress-related genetic variants influence downstream inflammatory responses, thus identifying potential therapeutic targets.

Table 1 summarizes the genetic networks in AMD pathogenesis, which is adapted from Bhumika et al. (2024) [31].

## 6. What Is Next for AMD? Future Directions: Personalized Medicine and Targeted Therapies

As every science is multidisciplinary, bioinformatics and the use of artificial intelligence models are quickly molding into the world of this pathology, enabling the possibility of studying what was limited or restricted in the known molecular techniques. Different authors had successfully used bioinformatics tools to expand the veiled knowledge of AMD pathogenesis and treatments. Nadeem et al., 2022, made use of bioinformatical techniques to identify genes associated with both versions of AMD, then combined genes from various subtypes of the pathology to reflect different stages of it. As a result, they identified several drug classes that can affect multiple genes that play a major role in AMD. These drugs include antidiabetics, antioxidants, and lipid-lowering agents. Regardless of their prescription, metformin and antioxidants displayed a strong association with AMD-risk genes [59].

Another interesting study was carried out by Cao et al. (2022), in which they wanted to see the potential role of traditional Chinese medicine against AMD. By screening 268 active ingredients, anti-AMD corresponded to 258 ingredient targets, combined with 2160 disease targets in AMD, and obtained 129 drug disease common targets. The molecular docking showed four of the potential active ingredients had good affinity against IL-6, TNF, VEGFA, and MAPK3 [60]. Pucchio et al. (2022) made an excellent summary of the bioinformatic studies related to AMD, where it can be clearly displayed that these tools can be applied to several fields regarding the pathology: from the disease characteristics to treatment decisions and risk factors [61].

Deep into these studies, we find that artificial intelligence (machine and deep learning) is being applied to a great number of diseases in the medical field, but in ophthalmology, AMD has been a priority [62]. There are several algorithms developed with the support of machine learning to study the progression of AMD; for example, the literature reports studies of the genotypes and color fundus images to predict the progression either of dry or wet AMD within the timing of 7 years, using a modified deep convolutional neural network. Also, machine learning has been useful to combine different features, such as demographic, environmental, and imaging, to create a risk-scoring system for the progression of neovascularization. Some investigations used sociodemographic clinical data combined with AMD scores, achieving an 86% of accuracy for the prediction of a 2-year incidence of late AMD, 67% for dry and wet AMD.

Authors that explore pathways to analyze time to late AMD use models that input a severity grade predicted by the neural network from fundus images and patient-related features such as age, smoking practices, and genotype. Moreover, there have been algorithms developed to predict progression for retinal atrophy for nonexudative AMD cases reaching a prediction accuracy for four years and longer [60,61,63].

As these technologies are new and need further studies, they are extremely promising for the early detection of AMD and such eye diseases. They are to become routine tests at some time, but for now, the cost is high, and there is a lack of equipment and doctors with the knowledge to apply them, especially in undeveloped countries. However, they are important to assess as they provide information that is not available to obtain from other techniques and, moreover, information that can change patients’ lives.

The integration of bioinformatics into AMD research holds promise for the development of precision medicine strategies. By leveraging AI and multi-omics data, it is now possible to classify AMD subtypes based on molecular signatures, enabling the identification of patients who may benefit from specific antioxidant or anti-inflammatory treatments. Furthermore, bioinformatics-guided drug repurposing has identified compounds, such as metformin and lipid-lowering agents, that may mitigate AMD progression by targeting oxidative stress and inflammation pathways.

Despite these advancements, challenges remain in translating bioinformatics discoveries into clinical applications. A major hurdle is the validation of computational predictions through experimental and clinical studies. Additionally, there is a need to develop standardized databases that integrate genetic, imaging, and biochemical data to improve the accuracy of disease prediction models.

To address the complexity of AMD at a molecular and cellular level, future research should leverage in vitro and in vivo models that better replicate the disease’s progression. Experimental approaches and computational tools can help bridge current gaps in the understanding of AMD pathogenesis, particularly in the interplay between oxidative stress, chronic inflammation, and genetic predisposition. While traditional cell cultures and animal models have provided valuable insights, they have limitations in fully mimicking the human retinal environment. Emerging methodologies in advanced experimental models to explore AMD mechanisms such as [28,38,41].

Retinal organoids and 3D cultures: These models derived from induced pluripotent stem cells (iPSCs) offer a more physiologically relevant system to study the effects of oxidative stress and inflammation in retinal pigment epithelium (RPE) dysfunction:

CRISPR-based gene editing: Targeted gene-editing approaches can be used to study the specific contribution of AMD-associated risk alleles (CFH, ARMS2/HTRA1, C3) and their role in immune dysregulation and oxidative stress resistance in RPE cells.

With the increasing availability of genomic, transcriptomic, and imaging data, computational approaches can significantly enhance our ability to predict disease risk, progression, and therapeutic responses. The computational and bioinformatics-driven research strategies have been proposed to refine AMD research [31,53,58]. For example, drug repurposing using AI algorithms. Recent studies have identified potential AMD therapies, such as metformin and lipid-lowering agents, through bioinformatics-driven drug screening. Further computational validation and clinical trials are necessary to assess their efficacy.

A major gap in current AMD research is the underrepresentation of non-European populations in genetic studies. Population-specific studies and personalized medicine approaches should be prioritized in future research [52,64,65,66]. Expanding genome-wide association studies (GWAS) to diverse ethnic groups to refine risk prediction models. Investigating epigenetic modifications and how environmental factors (e.g., diet, UV exposure, pollutants) interact with genetic predisposition.

Developing personalized treatment strategies that account for genetic ancestry, immune aging, and metabolic status to optimize therapeutic interventions.

## 7. Systematic Analysis of Key Findings in AMD Research

Age-related macular degeneration (AMD) has been extensively studied using genomic, transcriptomic, and computational approaches, with research focusing on genetic variations in complement regulation, oxidative stress, and angiogenesis. To gain a comprehensive understanding of AMD pathogenesis, we reviewed multiple studies employing experimental models, genetic analyses, and bioinformatics tools. Table 2 presents a comparative analysis of key findings, highlighting the methodologies used, major discoveries, and existing limitations in AMD research.

The studies reviewed provide significant insights into the genetic, molecular, and computational aspects of AMD pathogenesis. However, despite considerable advancements, critical gaps remain in the translation of genetic findings into effective therapeutic strategies and the integration of multi-omics approaches for a holistic understanding of AMD. Below, we discuss the key strengths and limitations of the reviewed studies.

### 7.1. Strengths of Current AMD Research Approaches

#### 7.1.1. Identification of Key Genetic Risk Factors

Genome-wide association studies (GWAS) and population-based studies have successfully identified major AMD-associated loci, such as CFH, ARMS2/HTRA1, and C3, which play critical roles in complement regulation, oxidative stress, and angiogenesis.

Studies such as Fritsche et al. (2016) and De Jong et al. (2021) provide functional validation of genetic variants, improving our understanding of how specific mutations influence inflammation and disease progression [12,35].

#### 7.1.2. Integration of Computational and AI-Driven Approaches

Machine learning and AI-driven bioinformatics tools have enhanced risk stratification and predictive modeling, as seen in studies like Nadeem et al. (2022) and Perepelkina et al. (2021), where AI models were used to predict AMD progression based on genetic and imaging data. Single-cell transcriptomics and proteomics emerge as powerful tools to map cellular interactions in the retina, offering high-resolution insights into AMD pathogenesis [59,64].

#### 7.1.3. Role of Oxidative Stress and Inflammation in AMD Progression

Experimental models, such as those by Datta et al. (2017) and Blasiak (2020), demonstrate that chronic oxidative stress and inflammation contribute to RPE dysfunction [24,41].

Studies testing antioxidants and complement inhibitors (e.g., Chen et al. (2021)) suggest potential therapeutic pathways to mitigate AMD progression [38].

#### 7.1.4. Advances in Therapeutic Target Identification

Research into complement inhibitors (anti-C3, anti-C5 drugs) has provided novel treatment approaches for AMD, though these require further clinical validation.

The exploration of senolytic drugs and anti-inflammatory agents targeting RPE senescence (Macchioni et al., 2021) suggests alternative therapeutic strategies [45].

### 7.2. Limitations and Research Gaps in AMD Studies

#### 7.2.1. Limited Ethnic Diversity in Genetic Research

GWASs heavily focus on European populations, limiting the generalizability of findings to non-European ethnic groups.

Studies such as Lumi et al. (2016) highlight the varying influence of genetic risk factors in different populations (e.g., CFH Y402H has a weaker association in East Asians) [67].

Expanding multi-ethnic genomic databases is essential to improve personalized risk prediction.

#### 7.2.2. Incomplete Understanding of Gene-Environment Interactions

While genetic studies explain up to 70% of AMD heritability, the role of environmental factors (e.g., smoking, diet, UV exposure) remains underexplored.

There is a lack of longitudinal cohort studies that integrate genetic and environmental risk factors to assess lifestyle modifications in AMD prevention.

#### 7.2.3. Translational Gaps in Therapeutic Applications

While genetic discoveries have improved risk prediction, few therapies directly target AMD genetic pathways. Complement inhibitors have shown promise, but their efficacy varies among patients, and some clinical trials have yielded mixed results. Research into CRISPR-based gene therapies is still in early stages and requires further refinement for clinical application.

#### 7.2.4. Lack of Standardized Multi-Omics Integration

Most studies focus on single-level analyses (genomics, transcriptomics, or proteomics), without integrating multi-omics data to establish a complete molecular network of AMD pathogenesis. Artificial intelligence-driven biomarker discovery remains underutilized for identifying novel therapeutic targets beyond CFH and ARMS2/HTRA1.

## 8. Conclusions

AMD remains a complex and multifactorial disease, primarily driven by oxidative stress, chronic inflammation, and genetic susceptibility. The interplay between ROS accumulation, RPE dysfunction, and immune dysregulation creates a vicious cycle that accelerates retinal degeneration. Although significant progress has been made in elucidating AMD pathophysiology, major challenges persist in developing targeted therapies that effectively slow or prevent disease progression. Current treatments, such as anti-VEGF agents for neovascular AMD, have improved visual outcomes but remain inadequate for many patients.

Future research should prioritize a multi-disciplinary approach integrating molecular biology, bioinformatics, and artificial intelligence to refine AMD diagnostics and treatment strategies. Advancements in precision medicine, including gene therapy, antioxidant-based interventions, and immune-modulating therapies, hold promise for more effective disease management. Moreover, addressing gaps in population-specific genetic studies and investigating the role of environmental factors in AMD susceptibility will be essential in developing personalized treatment approaches.

By fostering a deeper understanding of the molecular mechanisms driving AMD and leveraging emerging technologies, the field can move toward innovative and targeted therapeutic solutions, ultimately improving patient outcomes and preserving vision.

## Figures and Tables

**Table 1:** Tabular discussion of the specific genes involved in age-related macular degeneration (AMD).

Genes Associated with	
Complement System	CFH, C3, CFB/C2, CFI, C3, C9, CD46
Extracellular Matrix Proteins	TIMP3, MMP2, MMP9
Angiogenesis	VEGFA, TGFBR1
Lipid Metabolism	APOE, LIPC, CETP, ABCA1
Apoptosis	IER3, TNFRSF10A, CTRB2
AMD risk	ARMS/HTRA1 locus, PLEKHA ARMS/LOC387715 HTRA1

**Table 2:** Comparative analysis of key studies on AMD genetics, pathophysiology, and therapeutics.

Study	Objective	Method	Key Findings	Strengths	Limitations
Fritsche et al. (2016) [35]	Identify genetic risk loci for AMD	GWAS meta-analysis (European cohort)	Identified 34 AMD-associated loci, including *CFH*, *C3*, *ARMS2/HTRA1*	Largest GWAS study in AMD	Lacks ethnic diversity
Lumi et al. (2016) [67]	Assess *CFH* and *ARMS2* risk in Chinese populations	Case–control genetic association study	Found weaker CFH Y402H association and stronger ARMS2 link in East Asian populations	Population-specific insight	Requires replication in multi-ethnic cohorts
De Jong et al. (2021) [12]	Functional analysis of complement system genes	Genomics and protein analysis	*CFH rs1061170* impairs complement regulation, promoting inflammation	Functional validation of genetic risk	Limited environmental interactions
Merle et al. (2023) [58]	The “10q26 enigma” (*ARMS2/HTRA1* locus) in AMD	Genomic and epigenetic analysis	*HTRA1* overexpression linked to choroidal neovascularization; *ARMS2* regulates oxidative stress	Provides epigenetic data on genetic associations	Requires in vivo validation
Pappas et al. (2021) [57]	Investigate protective haplotypes in AMD	Population genomics	*CFH-CFHR5* protective variants reduce AMD risk in some populations	Highlights variability in genetic risk	Needs functional validation
Datta et al. (2017) [41]	Role of oxidative stress in RPE degeneration	In vitro RPE oxidative stress model	ROS accumulation leads to mitochondrial dysfunction and chronic inflammation in AMD	Controlled oxidative damage study	Lacks in vivo validation
Blasiak (2020) [24]	Investigate senescence-associated inflammation in AMD	Molecular pathways analysis	Senescence-associated secretory phenotype (SASP) drives inflammation and photoreceptor loss	Links oxidative stress to inflammation	No direct experimental validation
Chen et al. (2021) [38]	Test antioxidant protection in AMD models	In vitro and in vivo retinal oxidative stress models	Fucoxanthin reduces oxidative damage in AMD models	Demonstrates therapeutic potential	Limited to early AMD
Nadeem et al. (2022) [59]	AI-based gene identification in AMD	Bioinformatics and machine learning	Identified key genes linked to oxidative stress and inflammation in AMD	High-throughput analysis	Requires experimental validation
Perepelkina et al. (2021) [64]	AI prediction of AMD progression	Machine learning on fundus images	AI models predict AMD severity and risk progression	High accuracy in clinical settings	Limited generalizability across populations
Macchioni et al. (2021) [45]	Investigating inflammation in aging retina	Pro-inflammatory cytokine analysis in RPE cells	Chronic inflammation accelerates AMD progression	Strong correlation with clinical AMD	Requires further patient validation
